# Chemopreventive Targeting of Oncogenic Stemness: EGCG-Mediated Suppression of the HIF-1α-PPARγ-Cancer Stem Cells Transcriptional Signature in 3D Glioblastoma Spheroids

**DOI:** 10.7150/jca.124247

**Published:** 2025-10-20

**Authors:** Abdallah Fallah, Maellis Payet-Desruisseaux, Alain Zgheib, Bogdan Alexandru Danalache, Nicoletta Eliopoulos, Borhane Annabi

**Affiliations:** 1 Laboratoire d'Oncologie Moléculaire, Département de Chimie, Université du Québec à Montréal, Montreal, QC, Canada, H3C 3P8.; 2 Lady Davis Institute for Medical Research, SMBD-Jewish General Hospital, and Department of Surgery, McGill University, Montreal, QC, Canada, H3T 1E2.

**Keywords:** metabolic reprograming, cancer stem cells, 3D spheroids, HIF, glioblastoma, adipogenesis, EGCG, PPARG

## Abstract

**Background:** Preventing the emergence and persistence of cancer stemness represents a promising strategy to reduce tumor aggressiveness and therapeutic failure. Cancer stem cells (CSCs), which contribute significantly to therapy resistance, recurrence, and metastasis, are sustained in part by metabolic reprogramming that enhances survival and self-renewal under stress conditions.

**Methods:** To model the hypoxic core of solid tumors, three-dimensional (3D) glioblastoma (GBM) spheroid cultures were generated using human U87, U118, U138, and U251 cell lines and compared to their respective two-dimensional (2D) monolayer cultures. Total RNA was extracted, and gene expression was analyzed via RT-qPCR and targeted gene arrays. Transient gene silencing was performed using specific siRNAs, while pharmacological intervention involved treatment with (-)-Epigallocatechin-3-gallate (EGCG), a bioactive phytochemical derived from green tea. Adipogenesis was evaluated using Oil Red O staining.

**Results:** Compared to conventional 2D cultures, 3D spheroids exhibited elevated expression of hypoxia-inducible factor-1 alpha (HIF-1α) and upregulation of peroxisome proliferator-activated receptor gamma (PPARγ), identified through adipogenesis array screening. Adipogenic activity persisted in the 3D spheroid model, and EGCG treatment effectively suppressed the upregulation of *HIF-1α* and *PPARG* transcripts. This led to a significant downregulation of adipogenic genes (*CEBPD*, *FOXO1*, *BMP2*, *BMP7*) and CSC-associated markers (*CD44*, *PROM1*, *ABCB5*, *ABCG2*), accompanied by reduced spheroid growth.

**Conclusions:** These findings underscore EGCG's chemopreventive potential in disrupting early HIF-1α-mediated molecular pathways that reinforce GBM stemness. By targeting hypoxia-driven metabolic reprogramming, EGCG offers a dietary-based approach to modulate the CSC niche and potentially delay or prevent GBM progression. Moreover, the use of 3D spheroid models highlights their relevance in preclinical chemoprevention research, bridging the gap between simplistic 2D cultures and the complex biology of solid tumors.

## Introduction

Cancer stemness is a central driver of tumor resilience and treatment resistance, making it one of the most pressing therapeutic targets in oncology. Cancer stem cells (CSCs), a specialized subpopulation within tumors, possess the capacity for self-renewal, tumor initiation, evasion of conventional therapies, and promotion of recurrence and metastasis 1. These properties enable CSCs to regenerate tumors even after aggressive treatment, contributing to clinical relapse and poor patient outcomes 2, 3. Consequently, dismantling the molecular mechanisms that support CSCs survival, often involving metabolic plasticity, signaling pathways, and adaptive resistance, is increasingly prioritized in therapeutic strategies aimed at eradicating the root drivers of malignancy rather than merely reducing tumor burden 4. Recent approaches include combining stemness-targeted therapies with chemotherapy or immunotherapy, although achieving therapeutic efficacy while minimizing toxicity remains a significant challenge 5, 6. Overall, targeting cancer stemness holds the potential to redefine therapeutic success, from transient remission to durable disease control and, ultimately, long-term cancer prevention.

In solid malignancies such as glioblastoma (GBM), cancer stemness is increasingly recognized as a key contributor to therapeutic resistance and tumor recurrence. GBM stem cells (GSCs) exhibit remarkable phenotypic and metabolic plasticity, enabling dynamic transitions that promote survival under cytotoxic stress 7, 8. Among these, mesenchymal GSCs demonstrate greater resistance to conventional therapies compared to proneural subtypes 9. This functional heterogeneity is closely tied to differential metabolic dependencies, with distinct GSCs populations variably relying on glutamine, glucose, or lipid metabolism, factors that significantly influence treatment responsiveness 10-12. Under therapeutic pressure, GBM tumors exploit this metabolic flexibility to evade immune surveillance and repopulate, driving disease relapse and poor clinical outcomes.

Recent studies highlight metabolic rewiring in GBM, particularly within hypoxic poorly vascularized tumor regions. In these niches, tumor cells upregulate genes involved in lipid biosynthesis, storage, and transport 13, 14. Transcriptomic analyses reveal co-expression of adipogenic and stemness-associated markers in chemoresistant GBM populations 15, suggesting a functional link between these programs. Moreover, tumor cells can reprogram surrounding adipocytes or hijack adipogenic signaling pathways to support their growth and survival 16. These findings underscore the therapeutic importance of targeting metabolic plasticity and stemness-related pathways in GBM to overcome resistance and improve long-term patient outcomes.

Emerging evidence suggests that cancer cells can co-opt adipogenic transcription factors, such as peroxisome proliferator-activated receptor gamma (PPARγ) and CCAAT/enhancer-binding protein alpha (C/EBPα), to facilitate metabolic adaptation within the nutrient-deprived, stress-inducing tumor microenvironment (TME) 17, 18. This phenotypic mimicry enhances lipid uptake and utilization, promoting cellular resilience and survival. In rapidly proliferating solid tumors like GBM, hypoxic niches arise due to inadequate vascularization. Under these conditions, hypoxia-inducible factors (HIFs) are upregulated, triggering a broad transcriptional response that includes activation of adipogenesis-associated pathways 19, 20. This metabolic reprogramming contributes to chemoresistance by altering drug permeability, reinforcing antioxidant defenses, and reshaping key components of the TME 19, 21. To effectively study these adaptations and their therapeutic implications, experimental models that replicate hypoxia-induced metabolic and transcriptional dynamics, such as three-dimensional culture systems, are essential.

Three-dimensional (3D) spheroid cultures provide a physiologically relevant *in vitro* model that closely mimics the architecture, oxygen and nutrient gradients, and microenvironmental complexity of solid tumors 22. Unlike traditional two-dimensional (2D) monolayer cultures, 3D spheroids exhibit structural heterogeneity, including proliferative outer layers, quiescent intermediate zones, and necrotic cores, reflecting the spatial dynamics of intratumoral regions 23. The hypoxic core and associated nutrient gradients promote the expression of CSC-associated transcriptional markers such as SOX2, OCT4, PROM1 (CD133), and NANOG, enabling the study of CSC plasticity and behavior under tumor-like conditions 24. Additionally, spheroids replicate diffusion limitations and cell-cell interactions that contribute to therapeutic resistance, making them valuable for investigating CSC-specific drug evasion strategies and screening agents that disrupt stemness-associated resilience pathways 25.

In this study, 3D GBM spheroids were employed as a physiologically relevant platform to assess the chemopreventive potential of (-)-epigallocatechin-3-gallate (EGCG), a bioactive compound derived from dietary green tea. By targeting the emergence of adipogenic features and CSC-associated transcriptional signatures, EGCG modulated the transcriptional landscape of GBM cells under conditions that better reflect the clinical disease phenotype. This diet-derived intervention highlights the potential for nutritional compounds to influence tumor plasticity and suppress the acquisition of stemness and metabolic reprogramming, hallmarks of therapeutic resistance and tumor recurrence. Importantly, such biologically grounded approaches may inform the development of pharmacologic strategies with enhanced predictive validity, bridging the gap between preclinical screening models and clinical efficacy, and improving the likelihood of translational success.

## Materials and Methods

### Cell culture

Human GBM-derived cell lines U87, U118, U138, and U251, along with their respective culture media, were obtained from the American Type Culture Collection (ATCC; Manassas, VA, USA). Cells were cultured in medium supplemented with 10% fetal bovine serum (FBS), without the use of antibiotics. Cultures were maintained in a humidified incubator at 37 °C with 5% CO_2_, and kept subconfluent to preserve cellular integrity. Cells were passaged bi-weekly at a 1:2 ratio and used for experiments within a maximum of eight passages to ensure consistency and minimize phenotypic drift.

### Formation of 3D hanging drop spheroids

Three-dimensional (3D) spheroid formation was achieved using the hanging drop technique and low-attachment culture methods, as previously described 26. Briefly, 100 mm non-adherent petri dishes were used, with lids removed and 10 mL of sterile phosphate-buffered saline (PBS) added to the base of each dish to create a hydration chamber. Using a multichannel pipette, 40 μL droplets of cell suspension (at densities of 10,000 cells per drop) were carefully dispensed onto the inner surface of the inverted lid, ensuring adequate spacing to prevent droplet contact. Up to 50 droplets were placed per lid. The lid was then inverted over the PBS-filled chamber and incubated at 37 °C in a humidified atmosphere (5% CO₂, 95% humidity) for 5 days. Droplets were monitored daily under a microscope to assess spheroid formation. Following aggregation, spheroids were either analyzed directly or transferred to 96-well round-bottom plates (FALCON #351177) pre-coated with poly(2-hydroxyethyl methacrylate) [poly-HEMA] (P3932, Sigma-Aldrich, Oakville, ON, Canada) to prevent cell adhesion. Each well contained 250 μL of fresh complete medium to support continued growth and viability.

### Microscopy and analysis of spheroids

Cell morphology was examined using a Nikon Eclipse TE2000-U inverted microscope (Nikon A1, Melville, NY, USA). Phase contrast micrographs were captured with a Q Imaging QICAM-IR Fast 1394 Digital CCD camera, operated via NIH μManager software (National Institutes of Health, Bethesda, MD). For imaging in 96-well plates, an Agilent BioTek Cytation 5 cell imaging multimode reader equipped with 4x and 10x objectives was used to acquire bright-field images of entire wells, using Gen5 software. Quantitative analysis of spheroid size was performed using QuPath version 0.5.1, in combination with the Segment Anything Model (SAM) extension 27. This tool leverages deep learning algorithms to segment objects within images by identifying contours and morphological features, enabling precise and reproducible measurements.

### Oil Red O lipid staining

Neutral lipid staining was performed using Oil Red O on both monolayer cell cultures and 3D spheroids. Prior to fixation, cells were transferred to culture plates and rinsed twice with phosphate-buffered saline (PBS) to remove residual medium and minimize staining artifacts. Cells were then fixed with 4% paraformaldehyde (PFA) for 10-15 minutes at room temperature. Following fixation, samples were rinsed with PBS and subsequently with distilled water. Excess PBS was gently blotted to prevent dilution of the alcoholic staining solution and ensure optimal lipid visualization. A freshly prepared Oil Red O working solution, composed of three parts 0.5% stock solution in isopropanol and two parts distilled water (3:2), was filtered and applied to the samples, followed by a 15-minutes incubation at room temperature. After staining, excess dye was removed by gentle washing with distilled water. Throughout the procedure, samples were kept hydrated to preserve cellular morphology and ensure specific lipid staining without precipitation artifacts. Image acquisition was conducted using the Agilent BioTek Cytation 5 cell imaging system (BioTek), utilizing both bright field contrast and fluorescence modes, with Texas Red filter cube.

### Transient transfection and RNA interference

U87 cells were transiently transfected with small interfering RNA (siRNA) sequences using Lipofectamine-2000 transfection reagent (Thermo Fisher Scientific). Gene silencing was performed using 20 nM siRNA against HIF-1α (Hs_HIF1A_5 siRNA, SI02664053), PPARG (Hs_PPARG_2 siRNA, SI00071680), or a non-targeting scrambled control (AllStar Negative Control siRNA, 1027281), all synthesized by QIAGEN (Valencia, CA, USA) and annealed to form duplexes. The efficiency of gene silencing was evaluated by RT-qPCR, as described below.

### Total RNA extraction, cDNA synthesis, and real-time quantitative PCR

Total RNA was extracted from 2D monolayer cultures and 3D spheroids using TRIzol reagent, following the manufacturer's protocol (Life Technologies, Gaithersburg, MD, USA). Total RNA concentration and purity were assessed using a NanoPhotometer P330 (Implen). A total of 2 μg of RNA was reverse-transcribed into cDNA using the high-capacity cDNA reverse transcription kit (Applied Biosystems; Foster City, CA, USA). Quantitative PCR (RT-qPCR) was performed using SsoFast EvaGreen Supermix (Bio-Rad; 1725204), and gene specific primer sets purchased from QIAGEN. The following QuantiTect primer sets were used: PPARG (Hs_PPARG_1_SG QT00029841), DDIT3 (Hs_DDIT3_1_SG QT00082278), FOXO1 (Hs_FOXO1_1_SG QT00044247), VEGF (Hs_VEGFA_1_SG QT01010184), GLUT1 (Hs_SLC2A1_1_SG QT00068957), HIF-1α (Hs_HIF1A_1_SG QT00083664), GAPDH (Hs_GAPDH_2_SG QT01192646) and PPIA (Hs_PPIA_4_SG QT01866137). Gene expression analysis was conducted on a Bio-Rad CFX Connect Real-Time PCR Detection System using Bio-Rad CFX Manager Software version 3.0. Relative expression levels were normalized to two housekeeping genes, *GAPDH* and *PPIA*, and calculated using the standard 2^-ΔΔCq^ method.

### Human cancer stem cell and human adipogenesis profiler PCR arrays

Premade RT^2^ Profiler PCR arrays for Human Cancer Stem Cells (PAHS-176Z) and Human Adipogenesis (PAHS-049ZD) were purchased from QIAGEN and used according to the manufacturer's instructions. Briefly, genomic DNA was removed prior to reverse-transcription of 0.5 μg of total RNA using the RT^2^ First Strand Kit (QIAGEN, 330404). Each array plate was used to analyze one cDNA sample, prepared with the RT^2^ SYBR Green qPCR Mastermix (QIAGEN, 330502). Gene expression analysis of 84 target genes and internal controls was performed using the GeneGlobe Data Analysis Center (QIAGEN; https://geneglobe.qiagen.com/us/analyze), applying the standard 2^-ΔΔCq^ method for relative quantification. In figures, fold regulation values were reported: for upregulated genes, fold regulation equals fold change, for downregulated genes, fold regulation was calculated as 1/(fold change).

### *In silico* analysis of transcript levels in clinical GBM and low-grade glioma tissues

Gene Expression Profiling Interactive Analysis (GEPIA) was used to examine RNA sequencing expression data from glioblastoma (GBM, n = 163) and low-grade glioma (LGG; n = 251) tumor samples, compared to normal brain tissue (n = 207), using data sets from The Cancer Genome Atlas (TCGA) and the Genotype-Tissue Expression (GTEx) databases 28. GEPIA offers a suite of customizable analytical tools, including tumor/normal differential expression analysis, cancer type and pathological stage profiling, patient survival analysis, similar gene detection, correlation analysis, and dimensionality reduction (http://gepia.cancer-pku.cn/detail.php, accessed on June 28th, 2025). Differential gene expression analysis was performed using one-way ANOVA, with disease state (GBM, LGG, or normal) as grouping variable for box plot visualization.

### Prognostic value of *HIF-1α*, *CEBPD*, *BMP7, FOXO1, LPL, DDIT3,* and *PPARG* in GBM patients

The prognostic significance of mRNA expression levels of *HIF-1α*, *CEBPD*, *BMP7, FOXO1, LPL, DDIT3,* and *PPARG* in GBM patients was evaluated using the GEPIA web server 28. For each gene, expression data were retrieved from a comprehensive web-based database containing high-quality RNA sequencing datasets from GBM tissues. Overall survival analysis was performed using the log-rank test to assess the correlation between gene expression levels and patient outcomes.

### Statistical data analysis

Data are presented as mean ± standard deviation (SD) from three independent experiments, unless otherwise specified. Statistical analyses were performed using non-parametric tests: the Mann-Whitney U-test for comparisons between two groups, and the Kruskal-Wallis test followed by Dunn's post hoc test for comparisons involving three or more groups. A *p*-value < 0.05 (*) was considered statistically significant and is indicated in the figures.

## Results

**Figure 1:**
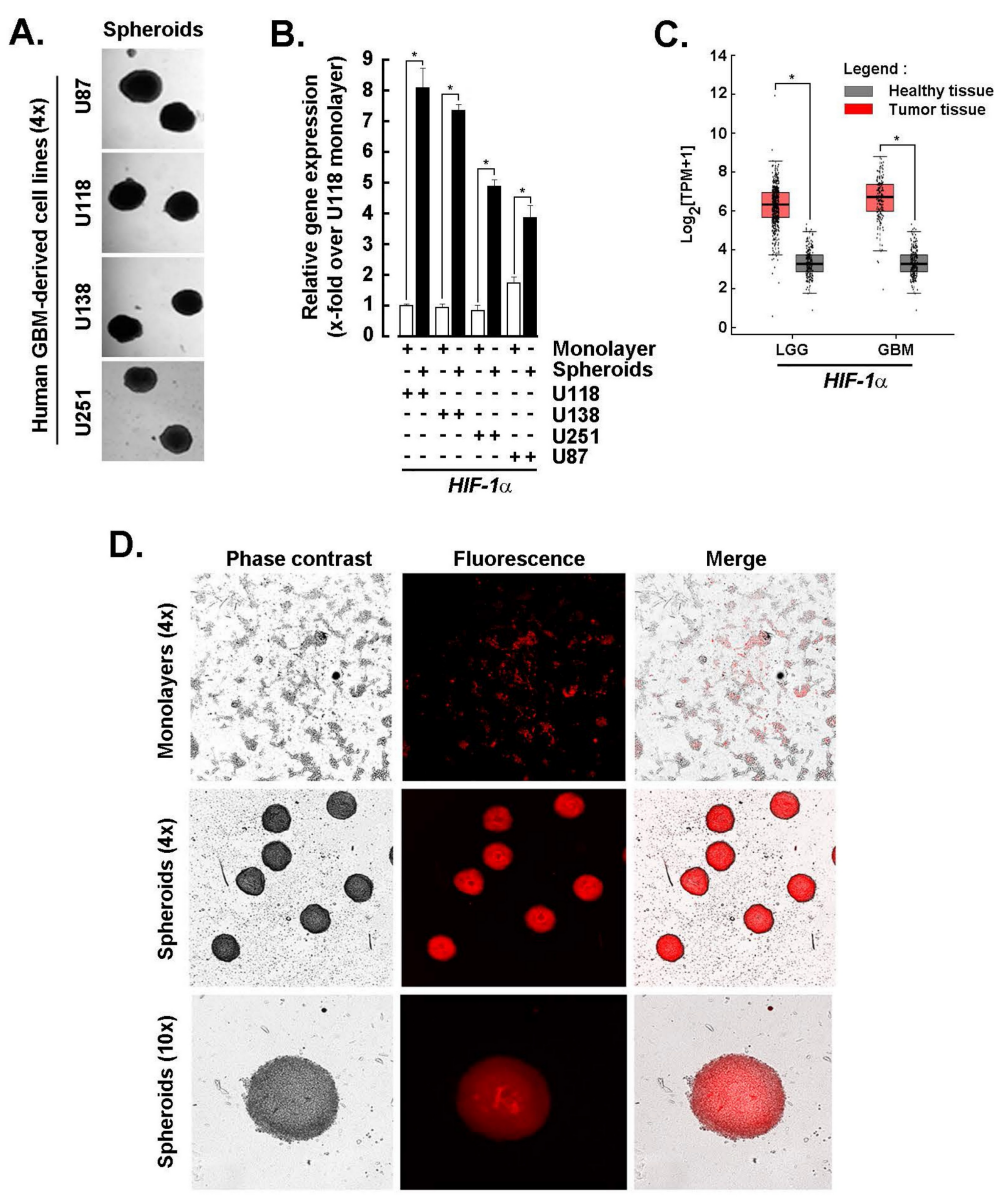
*Spheroids from GBM-derived cell lines share a high HIF-1α transcriptional signature with tumor tissues and exert active adipogenesis.* **A)** Monolayer cultures from human GBM-derived cell lines were used to generate 3D spheroids as described in the Methods section, and representative phase contrast pictures taken (Magnification = 4x). **B)** Total RNA was extracted from 2D monolayers (white bars) and 3D spheroids (black bars) of the indicated GBM-derived cells, and *HIF-1α* expression assessed by RT-qPCR as described in the Methods section (*P <0.05). **C)** *In silico* analysis of transcript levels was performed for HIF-1α using RNA extracted from clinical samples from GBM and low-grade glioma (LGG) (red boxes) and compared to healthy tissue (grey boxes) (*P <0.05). **D)** Active adipogenesis was assessed in 2D monolayers and 3D spheroids from U87 cells upon staining with Oil Red O as described in the Methods section.

**Spheroids from GBM-derived cell lines share a high *HIF-1α* transcriptional signature with clinical tumor tissues and maintain active adipogenesis.** Monolayer cultures derived from human GBM cell lines were used to generate 3D spheroids as described in the Methods section (**Fig. 1A**). Total RNA was extracted, and transcript levels of *HIF-1α*, a key regulator of tumor survival under hostile microenvironmental conditions, were assessed by RT-qPCR. Upon spheroid formation, *HIF-1α* expression was significantly upregulated in U87, U118, U138, and U251 cells (**Fig. 1B**, black bars), compared to their respective 2D monolayer counterparts (**Fig. 1B**, white bars). The coordinated cellular response is likely driven by hypoxia within the spheroid core, where HIF-1α orchestrates metabolic adaptation and vascular remodeling. A similar pattern of *HIF-1α* upregulation was observed in clinical samples of low-grade glioma (LGG) and GBM tumors compared to normal brain tissue (**Fig. 1C**), further validating the relevance of the spheroid model. Additionally, Oil Red O staining of U87-derived spheroids revealed prominent lipid accumulation, indicative of active adipogenesis and a shift toward a lipogenic metabolic phenotype (**Fig. 1D**). These findings support the presence of a hypoxia-induced molecular signature in GBM-derived 3D spheroids and prompt further investigation into how adipogenic adaptation contributes to the chemoresistant phenotype of CSCs.

**Figure 2:**
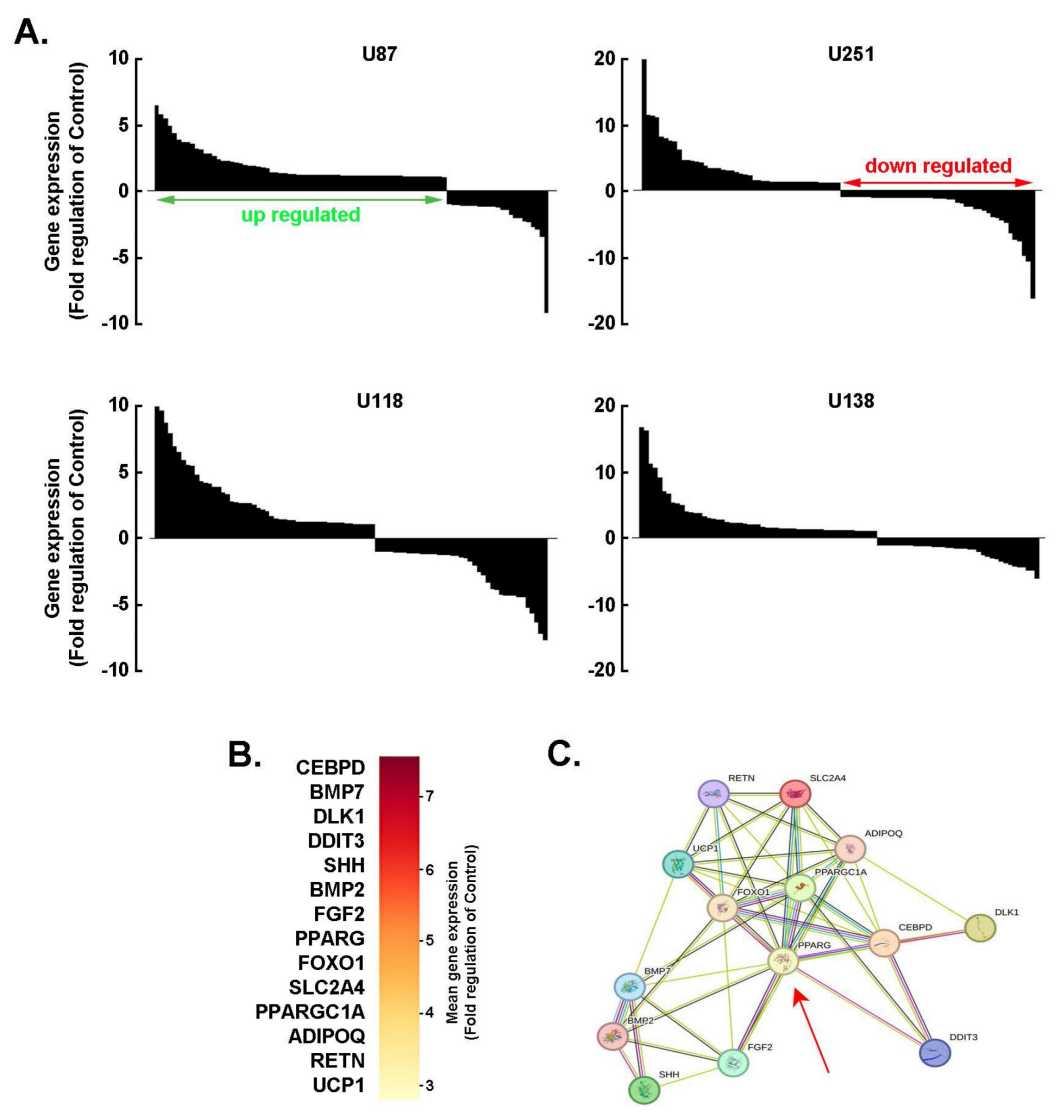
*PPARγ as a hub for the adaptive adipogenic transcriptional signature in 3D GBM-derived spheroids.* **A)** Total RNA was extracted from 2D monolayers or 3D spheroids of the indicated GBM-derived cells. Differential adipogenesis gene arrays was performed and representative fold-change histogram profiles of gene expression in spheroids cultures versus 2D monolayers shown. **B)** Heat map representation of the mean expression from the conserved top 14 genes upregulated upon spheroids formation from the four tested cell lines. **C)** Protein-to-protein interaction network of the 14 conserved and most upregulated gene expression triggered upon 3D spheroids formation as determined with STRING.

**PPARγ as a hub for the adaptive adipogenic molecular signature in 3D GBM-derived spheroids**. Investigating adipogenic adaptive metabolism in cancer cells is essential for understanding how tumors exploit lipid-related pathways to support growth, evade therapy, and reshape their microenvironment. By acquiring adipogenic traits, cancer cells gain metabolic flexibility, enabling them to switch between glucose and lipid utilization, thereby increasing their resilience to metabolic-targeted therapies. To explore this phenomenon, a screen of 84 adipogenesis-related genes was conducted across 3D spheroids generated from four human GBM-derived cell lines. The spheroids exhibited consistent patterns of gene upregulation and downregulation (**Fig. 2A**). Among the top 14 commonly upregulated genes were: *CEBPD*, an early adipogenic transcription factor that activates C/EBPα and PPARγ, *BMP7*, which promotes mesenchymal stem cells commitment to the adipogenic lineage, *BMP2*, which promotes adipogenic differentiation via SMAD signaling and PPARG activation, *PPARG*, a master regulator of adipogenesis that drives the expression of genes involved in adipocyte differentiation and lipid storage, and *PPARGC1A*, a coactivator of PPARγ (**Fig. 2B**). These genes were found to be functionally interconnected (**Fig. 2C**), with PPARγ emerging as a central hub linking multiple components of the adipogenic network (**Fig. 2C**, red arrow). Understanding this metabolic crosstalk opens new avenues for therapeutic intervention, such as targeting lipid metabolism or disrupting adipocyte-tumor signaling pathways, which may help overcome resistance and improve treatment outcomes in GBM.

**Figure 3:**
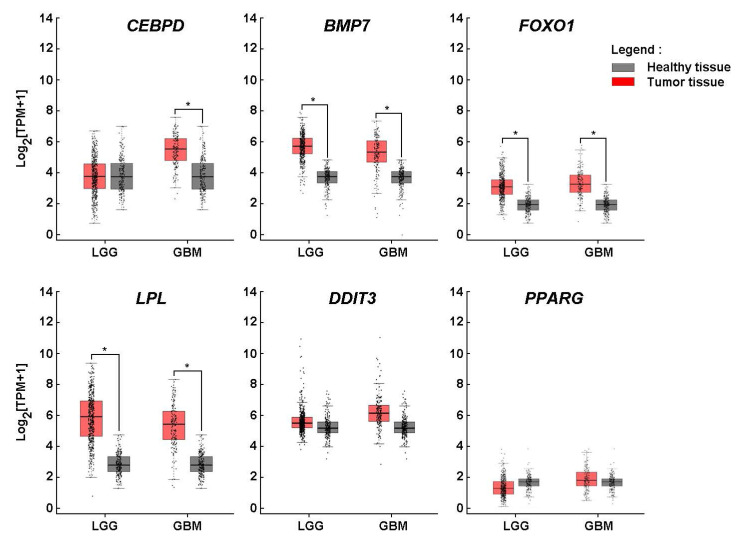
*Lipid metabolic adaptation in glioblastoma mediated by CEBPD, BMP7, FOXO1, and LPL overexpression*. *In silico* analysis of transcript levels was performed for *CEBPD*, *BMP7*, *FOXO1*, *LPL*, *DDIT3*, and *PPARG* using RNA extracted from clinical samples from GBM and low-grade glioma (LGG) (red boxes) and compared to healthy tissue (grey boxes) (*P <0.05).

**Lipid metabolic adaptation in GBM mediated by *CEBPD*, *BMP7*, *FOXO1*, and *LPL* overexpression.** The upregulation of *HIF-1α* and adipogenesis-related genes in 3D spheroids supports the hypothesis that GBM cells may activate adipogenic pathways to promote growth, survival, and therapy resistance. When gene expression analysis was extended to clinical tissue samples from low-grade glioma (LGG) and GBM, elevated levels of *CEBPD*, *BMP7*, *FOXO1*, and *LPL* were observed in both tumor types, except for *CEBPD*, which was not significantly upregulated in LGG, suggesting a metabolic reprogramming that favors adipogenic and lipogenic signaling. Interestingly, while these genes were induced upon spheroid formation, no significant differences in *DDIT3* and *PPARG* expression were detected in bulk tumor tissues compared to healthy controls (**Fig. 3**). This discrepancy may be attributed to tumor heterogeneity, particularly the variable expression of *PPARG* across GBM subtypes and cellular compartments. For instance, *PPARG* may be enriched in specific tumor cell populations, such as GBM stem-like cells, which are underrepresented in bulk tissue analyses. This differential interpretive approach underscores the importance of considering the distinct biological and genomic contexts in which these genes operate, and highlights the limitations of bulk tissue comparisons when investigating cell-type-specific regulatory mechanisms.

**Figure 4:**
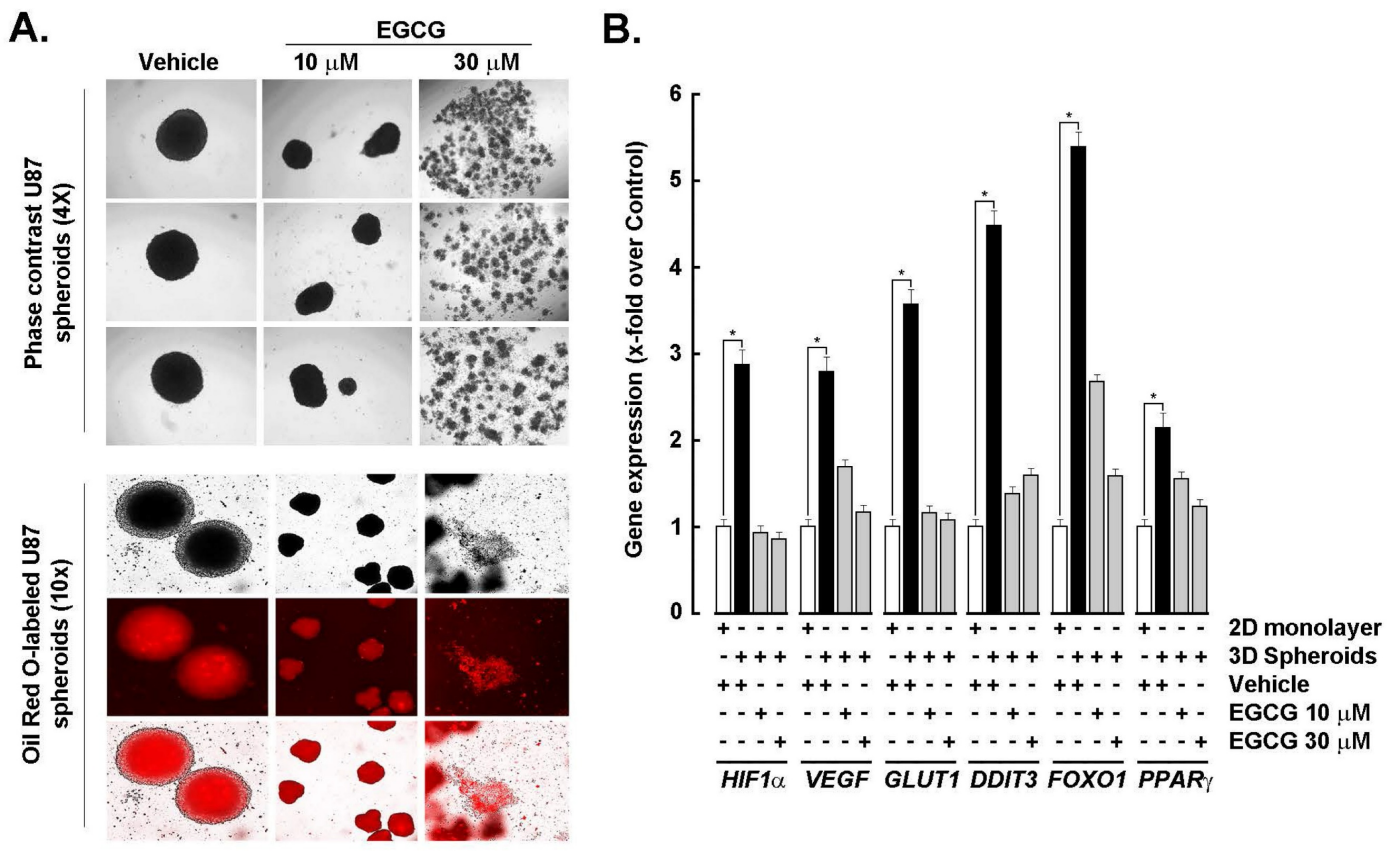
*EGCG inhibits HIF-1α and adipogenic signature in GBM spheroids.* **A)** Monolayer cultures from the human U87 GBM-derived cell line were used to generate 3D spheroids in the absence (vehicle) or presence of the indicated EGCG concentrations as described in the Methods section and representative phase contrast pictures taken (Upper panels; Magnification = 4x). Similarly, treated cells were stained with Oil Red O and representative phase contrast and fluorescent pictures taken (Magnification = 10x). **B)** Total RNA was extracted from 2D monolayers (white bars) and 3D spheroids (black bars) of the indicated GBM-derived cells and *HIF-1α*, *VEGF*, *GLUT1*, *DDIT3*, *FOXO1*, and *PPARG* expression assessed by RT-qPCR as described in the Methods section (*P < 0.05).

**EGCG transcriptional inhibition of *HIF-1α* and adipogenic signature in 3D GBM spheroids**. (-)-Epigallocatechin-3-gallate (EGCG), the predominant catechin in green tea, inhibits adipogenesis through multiple molecular mechanisms, making it a promising candidate for both anti-obesity and anti-cancer therapies. To investigate whether EGCG could modulate the transcriptional and signaling landscape associated with 3D spheroid formation, spheroids were generated from 2D monolayer cultures in the presence or absence of EGCG. EGCG treatment resulted in a dose-dependent reduction in spheroid size: 10 μM EGCG led to a noticeable decrease, while 30 μM disrupted spheroid integrity (**Fig. 4A**, upper panels). Concurrently, Oil Red O staining revealed diminished lipid accumulation in EGCG-treated spheroids, indicating suppression of active adipogenesis (**Fig. 4A**, lower panels). At the molecular level, EGCG dose-dependently inhibited the spheroid-induced upregulation of *HIF-1α*, as well as angiogenic markers *VEGF* and *GLUT1*, and adipogenic markers *DDIT3*, *FOXO1*, and *PPARG* (**Fig. 4B**). These findings suggest that EGCG interferes with hypoxia-driven metabolic reprogramming and adipogenic signaling, thereby disrupting the cellular adaptations that support GBM spheroid growth and stemness.

**Figure 5:**
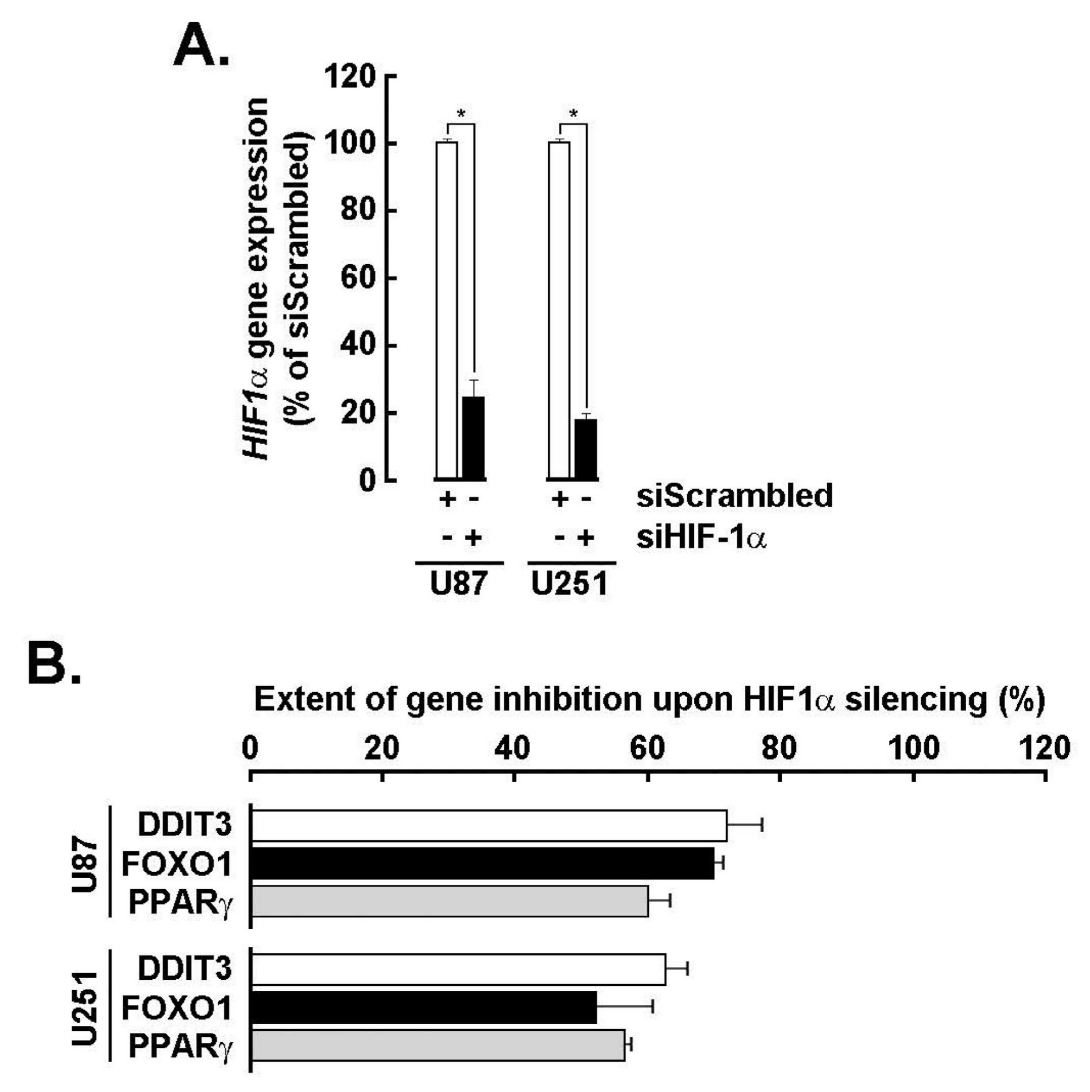
*HIF-1α transient silencing inhibits PPARG, DDIT3, and FOXO1 transcript levels*. **A)** Transient gene silencing of *HIF-1α* was performed in U87 and U251 cells as described in the Methods section and extent of gene silencing assessed by RT-qPCR (*P <0.05). **B)** Total RNA was extracted from U87 and U251 3D spheroids where *HIF-1α* was silenced, and impact on *DDIT3*, *FOXO1*, and *PPARG* expression assessed by RT-qPCR as described in the Methods section.

**Transient siRNA-mediated HIF-1α silencing inhibits the adipogenic signature in GBM spheroids**. To more specifically assess the role of HIF-1α in the acquisition of the adipogenic signature, transient gene silencing was performed in U87 and U251 cells (**Fig. 5A**). Knockdown of *HIF-1α* resulted in a marked reduction in the expression of adipogenic markers *DDIT3*, *FOXO1*, and *PPARG* in 3D spheroids (**Fig. 5B**), supporting its upstream regulatory role in driving adipogenic reprogramming. It is therefore reasonable to infer that the EGCG-mediated suppression of HIF-1α contributes to the downstream inhibition of these adipogenic effectors 29, further reinforcing the compound's potential to disrupt hypoxia-induced metabolic adaptation in GBM.

**Figure 6:**
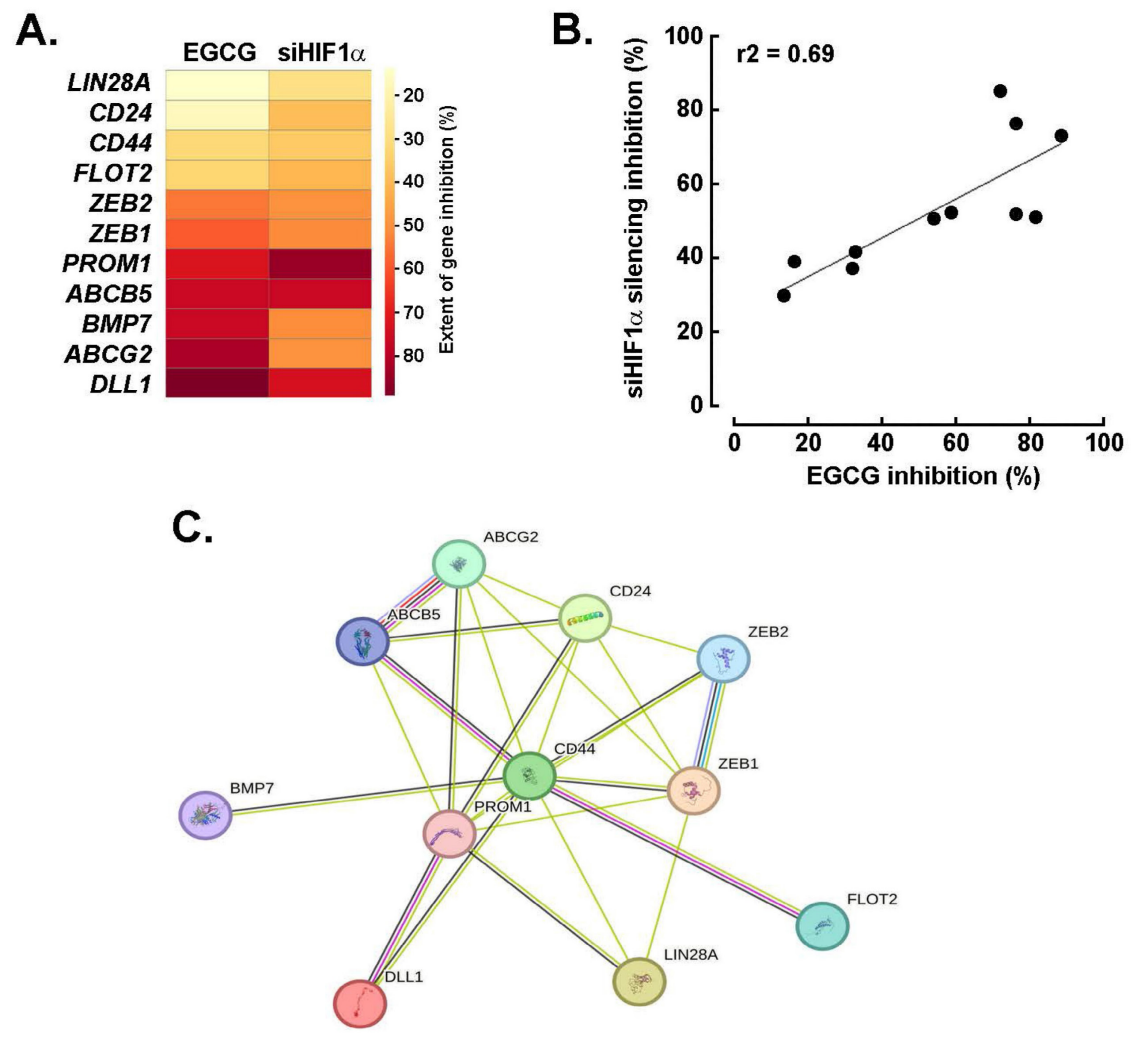
*EGCG and silencing of HIF-1α share a common transcriptional inhibition activity that impacts the acquisition of a CSCs transcriptional signature in U87 spheroids.* **A)** Representative heat map of the extent to which EGCG and* HIF-1a* silencing alters those eleven common genes induced expression and that characterize the acquisition of a CSCs transcriptional phenotype in spheroids. **B)** Correlation of the extent of inhibition of genes from A) between EGCG-treated cells and cells where *HIF-1α* was silenced. **C)** Protein-to-protein interaction network of the 11 most upregulated genes for which expression was altered by both EGCG and upon* HIF-1α* silencing in spheroids as determined with STRING.

**EGCG and silencing of HIF-1α share a common transcriptional inhibition activity that impacts the acquisition of a CSCs transcriptional signature in U87 spheroids.** To investigate the acquisition of a cancer stem cell (CSC) phenotype in 3D spheroids, gene expression profiling was performed using a targeted array. Among the genes found to be downregulated, both EGCG treatment and *HIF-1α* silencing inhibited the expression of 11 common targets, with the most significantly affected being *ABCB5*, *BMP7*, *ABCG2*, and *DLL1* (**Fig. 6A**). Interestingly, the extent of inhibition across these shared targets was well correlated, ranging from 20 to 80% reduction in expression (**Fig. 6B**). Further analysis using the STRING database confirmed coherent interrelationships among these genes, highlighting functional connectivity within the CSC-associated network (**Fig. 6C**).

**Figure 7:**
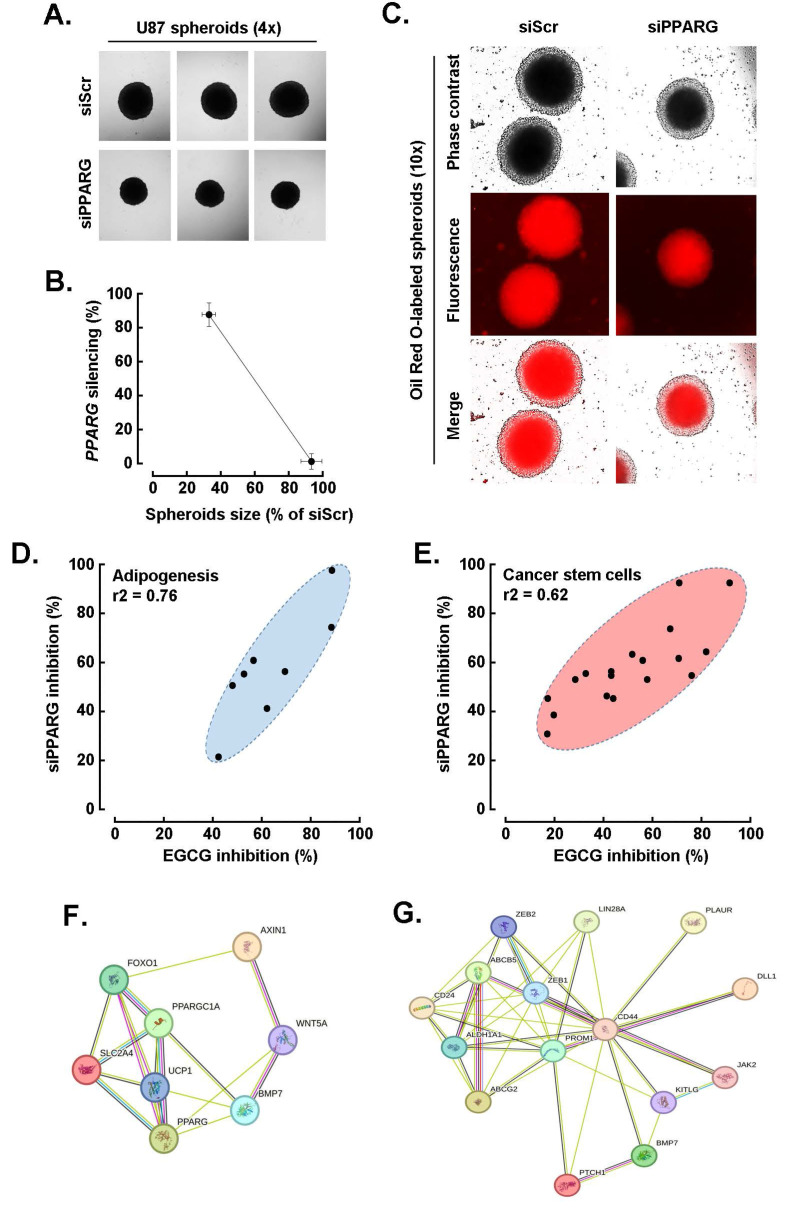
*EGCG targeting of PPARG alters both the cancer stem cell and adipogenic transcriptional signature in U87 spheroids.* **A)** Transient *PPARG* silencing was performed in monolayer cultures from human U87 GBM-derived cell lines and 3D spheroids generated as described in the Methods section, and representative phase contrast pictures taken and compared to control (siScrambled-transfected cells, siScr; Magnification = 4x). **B)** Correlation between 3D spheroids size and extent of *PPARG* gene silencing. **C)** Similarly, treated cells were stained with Oil Red O and representative phase contrast and fluorescent pictures taken (Magnification = 10x). **D)** Modulation of the top eight adipogenesis-related genes for which induction upon spheroids formation was inhibited by both EGCG treatment and *PPARG* silencing. **E)** Modulation of the top fifteen CSC-related genes for which induction upon spheroids formation was inhibited by both EGCG treatment and *PPARG* silencing. **F)** Protein-to-protein interaction network of the eight most upregulated adipogenic genes, and of **G)** the fifteen most upregulated CSC genes, for which expression was altered by both EGCG and upon *PPARG* silencing in spheroids as determined with STRING.

**EGCG targeting of *PPARG* alters both the CSCs and adipogenic transcriptional signature in U87 spheroids.** Building on the approach used for HIF-1α, we next investigated the interplay between EGCG and PPARγ in regulating the CSC and adipogenic transcriptional phenotype induced during U87 spheroid formation. To this end, *PPARG* was transiently silenced, and its impact on spheroid formation was assessed. Representative phase contrast images (**Fig. 7A**) revealed a significant reduction in spheroid size following *PPARG* knockdown, which correlated with the extent of gene repression confirmed by qPCR (**Fig. 7B**). Additionally, Oil Red O staining showed a peripheral reduction in lipid accumulation in *PPARG*-silenced cells, indicating suppressed adipogenesis (**Fig. 7C**). Expression analysis of the top eight adipogenesis-related genes, including *PPARG*, *FOXO1*, *SLC2A4*, and *BMP7*, demonstrated a consistent pattern of inhibition following either EGCG treatment or *PPARG* silencing, with a strong correlation (**Fig. 7D**, r2 = 0.76). A similar trend was observed for the top fifteen CSC-related genes, such as *PROM1*, *ABCG2*, *ABCB5*, and *CD44*, which also showed correlated downregulation under both conditions (**Fig. 7E**, r2 = 0.62). STRING network analysis further revealed functional crosstalk among the modulated adipogenesis-related genes (**Fig. 7F**) and CSC-associated genes (**Fig. 7G**), highlighting the interconnected transcriptional landscape influenced by PPARγ and EGCG during spheroid formation.

## Discussion

Glioblastoma (GBM) is characterized by pronounced metabolic plasticity, enabling tumor cells to dynamically adapt to hostile microenvironmental conditions, such as hypoxia, nutrient deprivation, and oxidative stress. These metabolic adaptations drive extensive transcriptional reprogramming, reinforcing pro-survival and therapy-resistant phenotypes. To investigate the molecular interplay between metabolic shifts and stemness-associated transcriptional networks, we employed a 3D GBM spheroid culture system that recapitulates key features of *in vivo* tumor physiology. This model was used to examine the induction of adipogenic transcriptional signatures and their convergence with stem cell-associated gene expression programs, both of which contribute to chemoresistance and tumor persistence. Furthermore, we evaluated the modulatory effects of EGCG, a bioactive phytochemical derived from dietary green tea, on the coordinated transcriptional regulation mediated by HIF-1α and PPARγ. EGCG attenuated the expression of adipogenic and stemness-related markers, underscoring its potential as a chemopreventive agent capable of disrupting transcriptional and metabolic vulnerabilities in GBM. These findings support the development of integrative therapeutic strategies that simultaneously target metabolic flexibility and transcriptional resilience, with the goal of improving treatment outcomes and reducing tumor recurrence.

Enhanced adipogenic signaling within tumor cells promotes lipid accumulation, creating intracellular reservoirs that can sequester lipophilic chemotherapeutic agents, and reduce their cytotoxic efficacy 14. This lipid-enriched microenvironment also contributes to the stabilization of HIFs, which reinforce cancer stem-like phenotypes and further enhance therapeutic resistance 30. Notably, the ATP-binding cassette transporter G2 (ABCG2), a key efflux protein implicated in multidrug resistance, is significantly upregulated in 3D spheroids and cancer stem-like cells. Its expression is closely associated with adipogenic molecular pathways 31. Hypoxia-induced lipid accumulation observed in malignancies such as breast, ovarian, and GBM, correlates with sustained stemness and diminished treatment responsiveness. In parallel, adipokines such as leptin and interleukin-6, upregulated under hypoxic conditions, promote tumor progression and facilitate immune evasion 32. In solid tumors, hypoxic stress can induce adipogenic-like metabolic traits that critically influence tumor plasticity and drug resistance. Consistent with the inhibitory effects of EGCG on *PPARG* transcript levels, pharmacologic inhibition of PPARγ or lipid metabolic pathways has been shown to restore chemosensitivity in preclinical models 33. Collectively, these findings highlight the pivotal role of an adipogenic transcriptional program in remodeling the TME to favor chemoresistance. Moreover, the diet-derived phytochemical EGCG demonstrates potential in targeting adipogenic-like metabolic reprogramming, offering a promising chemopreventive strategy against transcriptionally driven therapy resistance in GBM.

Beyond metabolic reprogramming, CSC plasticity plays a critical role in shaping therapeutic outcomes by enabling resistance mechanisms, driving tumor recurrence, and promoting metastatic dissemination, each representing major barriers to sustained remission. CSCs exhibit intrinsic resistance to chemotherapy and radiotherapy, largely due to their quiescent state, enhanced DNA repair capacity, and elevated expression of drug efflux transporters 34. Additionally, CSCs also exert immunomodulatory effects within the TME, dampening host immune surveillance and compromising the efficacy of immunotherapeutic strategies. Conventional treatment modalities, which primarily target proliferative tumor cells, often fail to eliminate this resilient CSC population. As a result, therapeutic relapse remains a significant challenge. Our findings support the development of future therapeutic strategies aimed at disrupting stemness-associated signaling pathways or leveraging chemopreventive agents, particularly those derived from dietary sources, for their capacity to prevent the acquisition of CSC characteristics and enhance treatment responsiveness 35.

Targeting CSC plasticity represents a promising therapeutic strategy to overcome treatment resistance and improve clinical outcomes. Approaches aimed at disrupting epithelial-mesenchymal transition (EMT) and reversing stemness-associated traits, particularly through inhibition of key transcriptional regulators such as SNAIL, ZEB, and TWIST, have gained traction for their potential to reduce tumor aggressiveness and therapy resistance 36. Diet-derived phytochemicals, including curcumin, resveratrol, sulforaphane, and EGCG, have demonstrated the ability to inhibit EMT and downregulate these transcription factors, thereby attenuating CSC-related phenotypes and enhancing sensitivity to conventional therapy 37-39. Beyond direct effects on cancer cells, modulation of the CSC niche, including immune infiltrates, cancer-associated fibroblasts, and extracellular matrix components, can further influence stemness dynamics. For example, resveratrol and sulforaphane have shown efficacy in suppressing HIF-1α activity and reprogramming immune responses, thereby compromising CSC survival and reducing tumor persistence 40. Differentiation therapy is another emerging approach, wherein agents such as retinoic acid and genistein promote the transition of CSCs into non-stem-like phenotypes that are more susceptible to standard treatments 6, 41. Collectively, these strategies underscore the multifaceted potential of combining phytochemical-based interventions with conventional therapies to effectively target CSC-driven resistance and prevent tumor relapse.

The integration of diet-derived phytochemicals into pharmacological studies using 3D GBM spheroid models represents a significant advancement in preclinical therapeutic research. Unlike conventional 2D monolayer cultures, 3D spheroid systems more accurately recapitulate the TME, including spatial gradients of oxygen, nutrients, and drug diffusion. These features enhance the physiological relevance and predictive validity of experimental outcomes. Emerging preclinical evidence suggests that phytochemicals such as EGCG interfere with adipogenic transcriptional pathways mediated by PPARγ and C/EBPα, which contribute to metabolic reprogramming and the stabilization of stem-like phenotypes in GBM. This dual impact, on both CSC-associated signaling and adipogenic transcriptional programs, offers a novel therapeutic strategy, particularly within 3D spheroid systems that mimic key aspects of *in vivo* tumor biology. Such models not only improve translational relevance but may accelerate the identification of effective chemopreventive or combination treatment regimens targeting metabolic and stemness-linked vulnerabilities in GBM.

**Figure 8:**
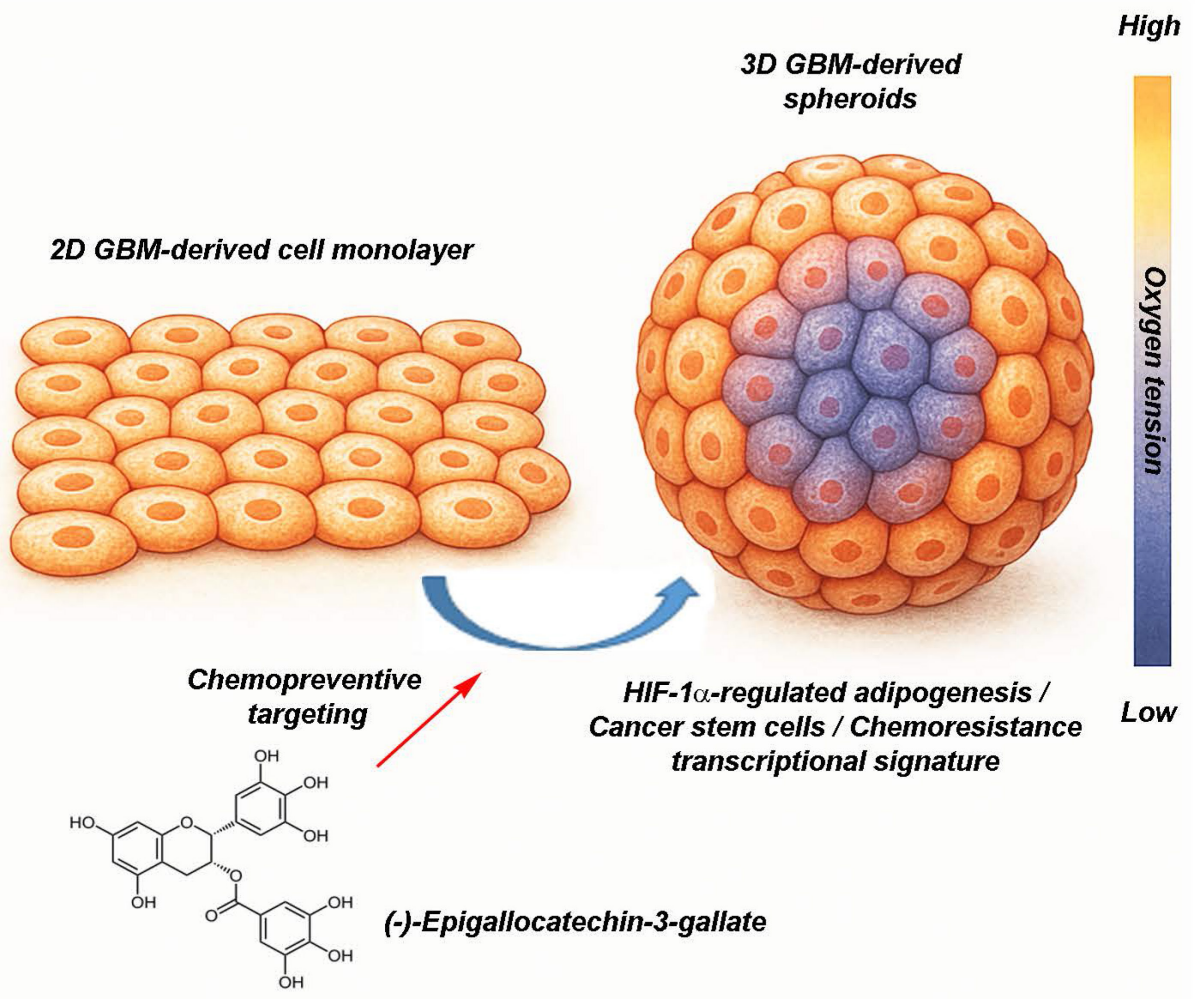
*EGCG targets 3D spheroids by disrupting hypoxia-driven processes, cancer stem cells, and adipogenesis-mediated resistance-linked transcription.* This figure presents two* in vitro* culture models of GBM, a classical 2D cell monolayer and a more physiologically relevant 3D spheroid culture. The 3D spheroid model better mimics the TME, particularly through the presence of an oxygen gradient, with normoxic conditions at the periphery and hypoxia toward the core. This spatial heterogeneity enables more accurate assessment of tumor behavior and treatment response. The diagram also illustrates the chemopreventive property of (-)-Epigallocatechin-3-gallate (EGCG), a green tea-derived polyphenol, which targets key pathways linked to metabolic rewiring and tumor chemoresistance. Specifically, EGCG is depicted as inhibiting HIF-1α-regulated adipogenesis, and modulating transcriptional profiles associated with cancer stem cells and therapy resistance. These insights underscore the necessity of using complex 3D models in preclinical research and highlight EGCG as a promising candidate in overcoming GBM treatment challenges.

GBM also remains one of the most treatment-resistant forms of brain cancer, largely due to the overexpression of ATP-binding cassette transporters such as ABCG2. These transporters actively efflux chemotherapeutic agents from cancer cells, thereby reducing intracellular drug concentrations and diminishing therapeutic efficacy. In this context, EGCG has shown promising potential to overcome drug resistance. Clinically, EGCG is recognized for its ability to sensitize GBM cells to chemotherapy, particularly temozolomide, by downregulating drug efflux mechanisms, enhancing intracellular drug retention and increasing cytotoxicity in GSCs. At the molecular level, EGCG modulates ABCG2 expression and function through multiple pathways. It suppresses transcription factors such as NFκB and HIF-1α, both of which are known to upregulate ABCG2. Additionally, EGCG exerts epigenetic effects, including alterations in DNA methylation and histone acetylation, which may contribute to the silencing of *ABCG2* gene expression. EGCG has also been shown to reduce stemness markers and impair neurosphere formation, indirectly suppressing ABCG2 levels in CSC populations. Moreover, EGCG induces oxidative stress and apoptosis in GBM cells, disrupting survival pathways that support ABCG2 expression. Together, these molecular insights, summarized in **Fig. 8**, underscore EGCG's potential as an adjunctive therapy aimed at improving treatment outcomes in GBM by targeting multidrug resistance mechanisms.

## Conclusions

This study underscores the value of physiologically relevant 3D GBM spheroid models as platforms for investigating the molecular mechanisms underlying tumor plasticity and therapeutic resistance. By integrating the use of EGCG, a diet-derived phytochemical, we demonstrate its potential as a chemopreventive agent capable of disrupting tumor-promoting transcriptional programs. Specifically, EGCG attenuated the induction of a hybrid adipogenic/CSC transcriptional signature, which is associated with tumor cell adaptation to hypoxic and nutrient-deprived microenvironments. These findings suggest that compounds such as EGCG may serve not only as adjuncts to conventional therapies, but also as modulators of tumor cell resilience by targeting key metabolic and stemness-associated pathways. Importantly, this chemopreventive strategy aligns with the broader concept of leveraging nutritional compounds to inform precision medicine approaches and enhance therapeutic efficacy in GBM. Future translational efforts may benefit from combining EGCG with agents specifically designed to eradicate CSCs, potentially overcoming treatment resistance and reducing tumor recurrence.

## Abbreviations

ATCC: American type culture collection
C/EBPα: CCAAT/enhancer-binding protein alpha
CSC: Cancer stem cells
EGCG: (-)-Epigallocatechin-3-gallate
EMT: Epithelial-mesenchymal transition
FBS: Fetal bovine serum
GEPIA: Gene expression profiling interactive analysis
GBM: Glioblastoma
GSC: Glioblastoma stem cells
GTEx: Genotype-tissue expression
HIF-1α: Hypoxia inducible factor-1 alpha
LGG: Low-grade glioma
PBS: Phosphate-buffered saline
PPARG: Peroxisome proliferator-activated receptor gamma
siRNA: Small interfering RNA
TME: Tumor microenvironment
